# Implementation of a Recuperation Unit and Hospitalization Rates Among People Experiencing Homelessness With COVID-19

**DOI:** 10.1001/jamanetworkopen.2021.2826

**Published:** 2021-03-10

**Authors:** Joshua A. Barocas, Mam Jarra Gai, Laura F. White, Deanna Faretra, Kerry Sachs, Miriam Komaromy

**Affiliations:** 1 Section of Infectious Diseases, Boston Medical Center, Boston, Massachusetts; 2 Department of Medicine, Boston University School of Medicine, Boston, Massachusetts; 3 Grayken Center for Addiction, Boston Medical Center, Boston, Massachusetts; 4 Department of Biostatistics, Boston University School of Public Health, Boston, Massachusetts; 5 Strategy Team, Boston Medical Center, Boston, Massachusetts; 6 Section of General Internal Medicine, Boston Medical Center, Boston, Massachusetts

## Abstract

This cohort study examines the association of the opening of a COVID-19 recuperation unit adjacent to a large safety-net hospital with COVID-19 hospitalization rates among people experiencing homelessness.

## Introduction

Coronavirus disease 2019 (COVID-19) may spread rapidly through homeless shelters because they are congregate settings^1,2^ where factors associated with the spread of the virus are common, including lack of personal protective equipment, overcrowding, and lack of recuperation spaces. As Boston experienced a COVID-19 surge that disproportionately affected persons experiencing homelessness (PEH) and threatened to overwhelm hospital capacity, a large safety-net hospital implemented a novel COVID-19 Recuperation Unit (CRU) for this population. The CRU, located adjacent to Boston Medical Center (BMC), provided isolation and quarantine for PEH and treatment for substance use.^3^ We aimed to determine the association of the care provided by the CRU with COVID-19 hospitalizations among PEH.

## Methods

We analyzed the daily COVID-19 hospitalization census from March 2020 to June 2020 at BMC, New England’s largest safety-net hospital. We included COVID-19–related hospitalizations (inpatient and observation) of adults aged 18 years or older, and excluded surgical and obstetrical patients. COVID-19 positivity was defined as (1) COVID-19 as primary diagnosis, (2) documented positive COVID-19 reverse transcriptase–polymerase chain reaction result, or (3) classification as COVID-19 positive by hospital infection control. We defined PEH as individuals who screened positive for homelessness or housing instability at intake.

Data were analyzed in October and November 2020. We used an interrupted time series with the opening of the CRU on April 9 as the interruption. The outcome was the rate of PEH in the hospital, modeled with a Poisson regression model that included terms for homelessness, intervention, secular time trends, and interaction terms for these patient characteristics with the daily hospital census (ie, the denominator of the rate) as an offset term in the model. This project was approved with a waiver of informed consent by the Boston University Medical Campus institutional review board because deidentified data were used. The study followed the Strengthening the Reporting of Observational Studies in Epidemiology (STROBE) reporting guideline.

## Results

From March 1 to June 4, 226 people were admitted to the CRU; 163 patients (72%) were men, and 88 patients (38.9%) were non-Hispanic Black. During that time, 8864 total patients were admitted to BMC, of which 1081 (13.2%) were PEH. The daily hospitalized count of COVID-19 PEH before and after the CRU opened ranged from 2 to 28 patients and 1 to 25 patients, respectively; whereas for stably housed patients, counts ranged from 11 to 190 and 23 to 185, respectively (Figure). The total COVID-19 hospital census count increased until April 8 (210 patients), with the highest single day increase occurring from April 7 (181 patients) to April 8 (210 patients) (Figure). The daily census at the CRU ranged from a low of 8 patients on June 4 to a high of 74 patients on April 21 (median [interquartile range], 33 [19-41] patients). PEH accounted for 84% of the CRU census. Among COVID-positive PEH in the BMC system, there was a 28% reduction in hospitalizations after the CRU opened relative to before (risk ratio, 0.72; 95% CI, 0.63-0.82).

**Figure.**
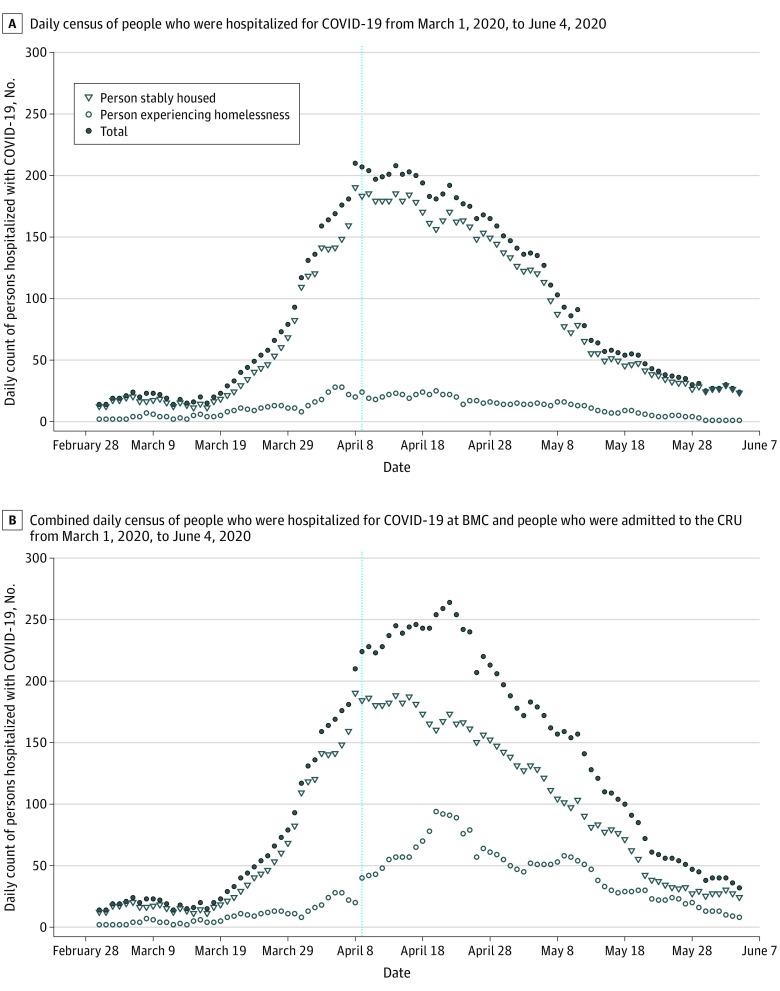
Daily Census of Hospitalization From Coronavirus Disease 2019 (COVID-19) at Boston Medical Center Figure A does not include the daily count of people at the CRU. Figure B shows the additive numbers of PEH in the hospital and the CRU. The CRU admitted people with confirmed COVID-19 from the Boston Medical Center inpatient unit and emergency department, other local emergency departments, and through surveillance testing at Boston-area shelters from April 9 (indicated by the vertical blue line) through June 4, 2020.

## Discussion

Our study demonstrates that the CRU was associated with a decrease in hospitalization among PEH with COVID-19. We used an interrupted time series design that provides evidence for an association of a novel CRU—deployed amidst the COVID-19 surge in Boston—with a decrease in COVID-19–related hospitalizations among PEH. The CRU provided a safe discharge location so that inpatient beds were used by patients who needed acute care, especially while COVID-19 prevalence in homeless shelters was high.^2^ It admitted patients who did not need hospitalization and those who were medically stable for hospital discharge but could not return to a shelter due to their inability to isolate.

A limitation of this study is that we may have missed hospitalizations elsewhere because we analyzed only BMC hospitalizations. However, because BMC is the primary hospital used by PEH in Boston, it is unlikely that many hospitalizations occurred elsewhere.

Our study adds to the growing body of evidence that supports the usefulness of alternative care sites for PEH and others who cannot return to a safe home. Investments in innovative approaches like CRUs may help limit the spread of COVID-19 among PEH by providing isolation and quarantine space and can help hospitals contend with COVID-19 surges.
